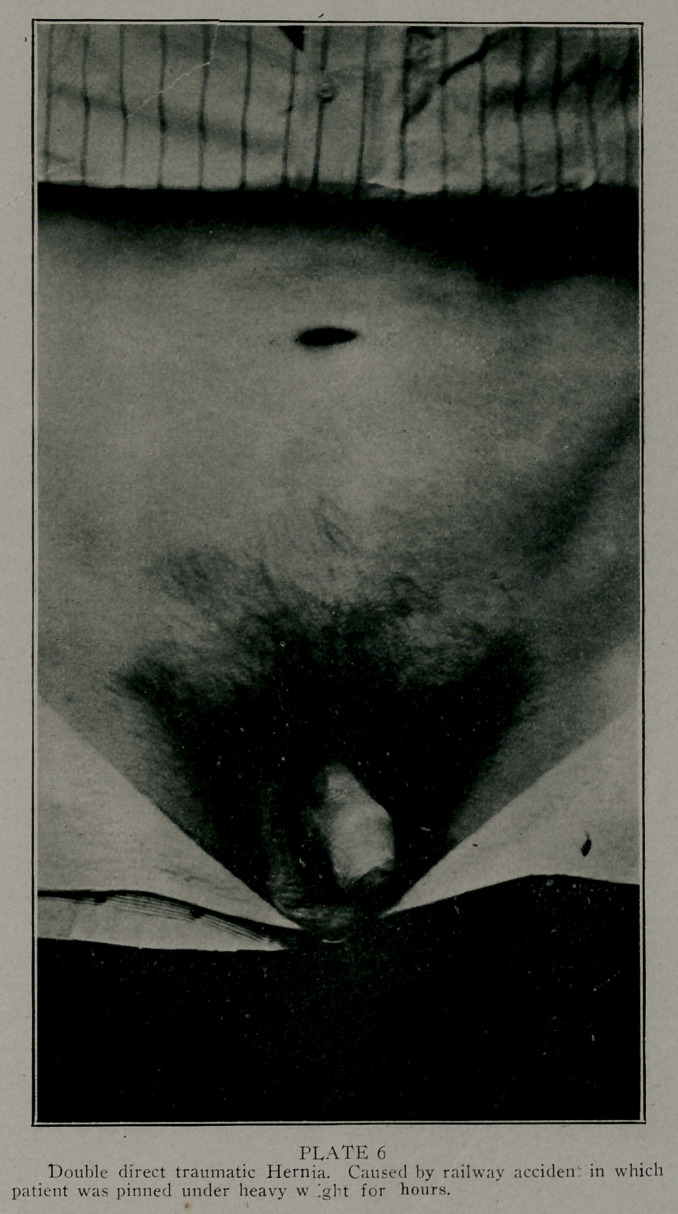# Report of One Hundred and Forty-Eight Herniotomies with Description of Operation

**Published:** 1914-01

**Authors:** L. C. Fischer

**Affiliations:** Atlanta, Ga.


### REPORT OF ONE HUNDRED AND FORTY-EIGHT HERNIOTOMIES WITH DESCRIPTION OF OPERATION.

By L. C. Fischer, M. D., Atlanta, Ga.

While a great deal has been said and written about hernia, there is no disease or abnormal condition which demands more attention and more careful consideration from both the surgeon and the medical man. The percentage of people suffering from this condition is great; varying as given by authorities from 1-16 to 1-8 of all the people, being much larger in men than in women. As given by statistics it is about 3 to 5. In my experience the percentage is much greater than this in men. In a series of 148 operations, only ten were upon women; four of these being strangulated. Of the total number of operations thirty were strangulated; the time of strangulation varying from a few to thirty-six hours. (Plate 4, showing the case that had existed for thirty-six hours.) It has been my misfortune to see four strangulated, where the condition of the patient was such that I could not operate; death occurring in all of these in less than twelve hours after first seeing them. There has been much discussion as to the classification of hernias. In a large proportion of cases, it is impossible before operation to classify them, especially, where there is much swelling or enlargement. As to the contents of the sac, I have found it impossible to tell this until it is opened. In my series I have found four direct; three of which were acute traumatic; the fourth had existed since childhood, though was primarily traumatic. (Plate 6 shows a double direct hernia). 22 of the cases were double. (Plate 1 is an extreme example). The youngest case operated upon was a babe of four months, which was strangulated; the oldest, which was also strangulated, a man of 87; the oldest female was 66. The great majority of them ranged from youths of iix to men of fifty, the greatest percentage from twenty to forty. This corresponds with the percentage as given by Ferguson, Degarmo and others.

PLATE I

Double sliding oblique inguinal Hernia. Contents of sac on right side, cecum, appendix, small intestines and omentum. On left side sigmoid and small intes ines.

While I do not propose to discuss the etiology, etc., of hernia, will report operating upon six members of one family; the father, four sons and one daughter. The five men were inguinal, the daughter being femoral. I am not in this series reporting the latter. Early in my professional career I began giving special attention and study to the various forms of hernia, and the numerous operations for the radical cure. And too, the mechanical treatments and so-called cures of these conditions. After years of experience, with much time and thought expended upon this subject, I have confirmed my first ideas. They were then and are still that all hernias are surgical. with few exceptions: and that the so-called mechanical treatments, trusses, etc, are dangerous appliances. Even though the truss under normal conditions may hold the rupture up, there are so many accidents that will force or allow the mass to come or be forced down, or out from under the truss. While it is a fact that all hernias are surgical conditions, and no matter how proficient we may become as surgeons, or how successful we may be as to the radical cure, there are a great many sufferers who will insist upon the mechanical treatment until some day they are found to be in desperate condition from strangulation. Or they will go on to old age and discomfort, when, in a large number of cases trusses can not be found that will support the hernia. This is due to the relaxation of the muscles. People afflicted with hernia form a great army of patients who seek relief. In the event the patient will not submit to the radical cure, we should not allow him to drift into the hands of quacks and charlatans; but should refer him to the best truss fitter we know. While I do not approve of truss wearing, these patients must have protection from strangulation or incarceration. Any patient wearing a truss is a chronic invalid and not in a position to live a normal life, and should be constantly under the Doctor's instructions. Then too, the work of fitting trusses often falls into the hands of the druggist and instrument shops; where as a rule there is very little knowledge of Anatomy or Pathology. Therefore where the patient will not have an operation, the responsibility of his care still falls upon us. That we are

PLATE 2
Large oblique scrotal Hernia on left with undescended testicle on right side.

in a large measure responsible for some of the conditions we meet due to a bad fitting truss, is true without question. Where the patient will wear a truss, we should give him instructions as to the care of his condition, viz.:—That the skin under the pad should have daily care, that the truss should fit well and hold the mass in position at all times; and that as soon as he suffers pain or discomfort he should report his condition at once. The injuries that he will suffer should also be explained, such as causing varicocele from pressure over the cord at the external ring, the weakening of the muscles under the truss, due to the support they receive; that the muscles are to a large extent fixed and not in action while the truss is on. But at the same time he must wear it all the time he is on his feet, that he will suffer some pain in the groins and back from pressure upon the nerves and the cord; especially is this true when the rings are large or the hernia hard to control. I have never tried to fit a truss for the reason that it is too much like trying to stop a leak in a mill dam by chinking the outside. Then, too, I have never seen one worn that it did not cause some pain and much discomfort, and at the same time the wearer is in constant danger of strangulation or incarceration of his hernia. While men are young, the muscles well developed, in a large majority of cases a truss fit by a competent man will hold the mass in the abdomen, provided there is no straining or unusual violence. But as age comes on, the muscles begin to lose their tone, a truss in many cases will not support the mass. Upon a recent trip into the country, I was impressed with this by seeing a very old man, ninety-two years of age, who when he walked, had to take both hands to hold up his hernia.

In a large percentage of cases where a truss has been worn, when they do come to operation, there is such a mass of adhesions formed around the cord and sac, that in a great many instances I have found it difficult to recognize the various tissues. In numbers of these the adhesions were so dense, it was a difficult task to dissect them apart without injury to important structures.

PLATE 3

Large oblique inguinal Hernia of right side with hydrocele Congential

In the preparation of patients for operation for hernia, the same care should be taken as for any other abdominal operation. If there is an infection in these cases, in almost every instance it means a failure. The matter of failure and recurrence is one of the stumbling blocks to medical men; they so often see their cases come back with "bad results," that they will hesitate to advise an operation. In a short paragraph by Dr. J. M. White, of Meridian, Miss., in which he advocates the non-operative treatment of inguinal hernia, he states: "If I were convinced that all operations for hernia always brought the two expanded muscles in freshened condition in complete apposition, I would retract all I have said about mechanical appliances." I quote this to show that the men who do not operate, realize that the muscles and fascias must be brought into complete apposition, that they must be clearly dissected and approximated without intervening tissues.

The safest time to operate upon hernia is when it is first recognized. Age and condition of patients, together with the surroundings are to be considered. A surgeon can never be too careful in his promises as to a permanent cure, for there are so many influences that may cause a recurrence; such as infected wounds, weak abdominal walls, poor general condition of the patient, being allowed to sit up too early, and finally, faulty formation of the rings, especially making them too large. (After operation for hernia, the patient should be confined to bed for at least fifteen days, and should not be allowed to do manual labor for thirty.)

All of these possibilities should be borne in mind and fully explained to the patient. In my series of 148 inguinal hernias, I have had two recurrences with no mortality. The two recurrences were in my first forty cases. They were due to infection. It was impossible for me to tell whether this was caused by suture material or faulty technique.

I have operated upon seven recurrences of other men. In these cases all of the normal anatomical relations had been changed. The adhesions from the previous operations made it difficult to recognize and get sufficient tissue to close the open-

PLATE 4

Large strangulated Hernia that had existed for thirty-six hours, it was not necessary to resect the gut.

ings as one would wish; all of these however were successful. There are men who would not do any other abdominal operation, who would attempt to cure hernia as though it was a very simple procedure. It is useless to dwell upon the anatomical relations, etiology, classifications, etc., as these are fixed facts. The importance of us knowing these relations and being able to recognize each of the tissues can't be exaggerated. Without the proper knowledge of the anatomy of the tissues and their relations, with which we have to deal, we, as well as the patient are doomed to disappointment. Considering carefully the causes of failures in the hands of good operators, I began to study them and immediately decided that there were several important ones. First, improper formation of the rings, second, that the muscles had not been properly denuded so that each part of these could be recognized, then too the proper disposition of the sac stump, or as I have read, by some operators, leaving the sac in position and not tying it at all. While studying these I began to use some other method of forming the rings, the disposition of the stump of the sac, and changing the relations of the cord. Up to this time I had done the Bazini and Feghuson operation. I do not claim any originality in this operation, but I had worked it out independent of others, using what I considered the good points of all other methods, making finally, one that in my work has proven successful. The operation as I have one it in a few more than one hundred of my series, is as follows:—The incision, the location of the various tissues, the dissection and ligation of the sac do not vary from that of any other radical cure. The disposition of the stump of peritoneum or sac, is one of the most important points of the operation. After ligating the sac, and cutting it off, the stump is fixed well up under the conjoined tendon and muscles that compose it in this manner: the two ends of the ligature with which the sac is tied are threaded into needles, then lifting well up the arching fibers of the internal oblique and transversallis muscles, the needle is passed from within outwards through the muscles, and the ends tied. This brings the stump well up under the muscles, and presents entirely new

PLATE 5

Ecarmans oblique scrotal Hernia, which had existed since childhood, would become encavcerated from slightest straining or undue exertion. was encarcerated at time of operation.

peritoneum at the original site of the rupture. Some of the best authorities have stated that in fomoral hernias especially, ligation of the sac is all that is necessary to make a complete cure. I do not agree with these radical ideas, at the same time, the care of the sac or stump means much. Should the sac be left undisturbed, even though the muscles are closed properly, there will almost certainly be a recurrence, due in most cases to the same forces that produced the original condition. The pressure of the abdominal contents will surely follow the old sac acting as a wedge, which will gradually enlarge the new formed rings. After disposing of the stump of the sac as above described, make a clean, distinct dissection of the cord and muscles, (taking great care to isolate the inguinal nerve and vessels), failure to do this properly is one of the greatest causes to allow or produce recurrences. All of the tissues in the inguinal region must be so dissected, that each individual one is recognizable. After this, retract the divided edges of the aponuerosis of the external oblique, together with the ilio-inguinal nerve, so that the conjoined tendon, and the muscles that compose it are well exposed both as to the insertion of the conjoined tendon into the crest and ilio-pectineal line of the ospubis, the arching fibers of the two muscles, also the origin of the muscles from Poupart's ligament. With the cord retracted well out of the way, close the conjoined tendon and the arching fibers of the internal oblique and transversallis, under the cord to the under margin of the gutter like process or portion of Poupart's ligament. Beginning at the insertion of these into the spine, crest and ilio-pectineal line, close them out to the original internal ring, then with the cord making its exit at this point, place two or three stitches external to the cord. This forms an entirely new ring. Failure to take these stitches above the cord or to the outer side of it, is liable to allow too large a ring to be formed. The origin of the muscles, from Poupart's ligament is largely muscular tissue, with very little tendonous attachment. While a great many operators lay much stress new ring thus formed has entirely new boundaries; the lower and upper ones being Poupart's ligament and the muscles above

PLATE 6

Double direct traumatic Hernia. Caused by railway accident in which patient was pinned under heavy w[illegible]ght for hours.

upon the muscular tissue, I am positive that the aponuerosis of the external oblique is the main stay in all radical cures. The mentioned, instead of simply coming through the transversallis fascia. To form the external ring and close the external oblique, begin at the same point, closing the upper cut edge of the aponuerosis of the external oblique, down to the same under margin of the gutter-like process of Poupart's ligament, out to the new formed internal ring, taking from one to three stitches external to the cord. Finally, close the lower cut margin of the aponuerosis of the external oblique up over the two rows of sutures, thus overlapping the aponuerosis of the external oblique, with the lower margin attached to the conjoined tendon, above the two lines of sutures. The same care should be taken to make allowance for the cord, or to complete the formation of the external ring. The overlapping is done in the same way as described by Halstead in his modification of Ferghuson's operation. This forms two entirely new rings, with one directly above the other, at the same time brings the cord out above at the original site of the internal ring. In this manner there is an entirely new floor formed for the cord, which is much stronger and safer than even the original one. Being made up now of the conjoined tendon and arching fibers of the muscles that compose it and a double layer of the aponuerosis of the external oblique. This closes entirely the external ring at its original site, which is the most dependent point of the abdomen. While the new rings are nearer the middle of Poupart's ligament, they are as far removed from the bony insertion of the muscles and ligaments as possible. Being placed as they are, there is more elasticity of the muscles at this point, making it possible to close more nearly the ring to the exact size of the cord; making it less likely to strangulate the cord, at the same time closing entirely the most dependent point of the abdomen. All of the recurrences that I have ever seen have been in the original course of the cord. One of the most important facts that guided me in this operation, or rather the adoption of it, is that I have never seen a recurrence in a female. The operation upon the female is much the same as above described, giving little heed to the round ligaments. In this operation I have never had a case of constriction of the cord, or odema of the testicle more than would be caused by the trauma of the operation. While with the external ring formed by the crest of the ospubis as its base, I have seen two cases of atrophy of the testicle due to the constriction of the cord. With the formation of the external ring as it was originally, its boundaries are Poupart's Ligament below, that portion of the aponuerosis of the external oblique that is attached to the crest and iliopectineal line above, with the crest of the ospubis as its base. In forming this ring there is so much danger of getting the opening too small or too large. The tendonous tissues at this point do not give or adapt themselves to the cord, which makes it necessary that the ring be left dangerously large. In this or any other operation for the radical cure of hernia, great care should be taken to not cut or injure the nerves that pass through the canal, or to include them in the ligatures; in either event it may cause atrophy of important muscles or great pain to the patient. With the above operation the cord is covered only by the fascias and skin. For a long time I was concerned for fear that this new course of the cord might cause pain in after life from it being now placed superficially and not as well protected as normally. But after doing the operation upon men in all walks of life, I have found that there was no cause for my fears. Suture materials used in these operations has been Kangaroo tendon formerly, but for the last two years have used either tanned or chromic gut with a continuous suture, using clips to close the skin. The wound should always be dressed with a spica bandage.

In operations for strangulated hernia, the procedure does not vary materially except that the suc should always be opened and its contents carefully examined before the constriction is cut.

Photographs by Edwards & Son, Atlanta, represent some of the largest of the series done by the method described. These patients are laborers, the last one of these was operated upon as much as a year ago. They are all well. The large double sliding hernia; I found the patient hauling tierces of lard weighing four hundred or more pounds in four weeks after the operation. This was much against my advice or wishes.

25 E. Linden Ave.

## Figures and Tables

**PLATE 1 f1:**
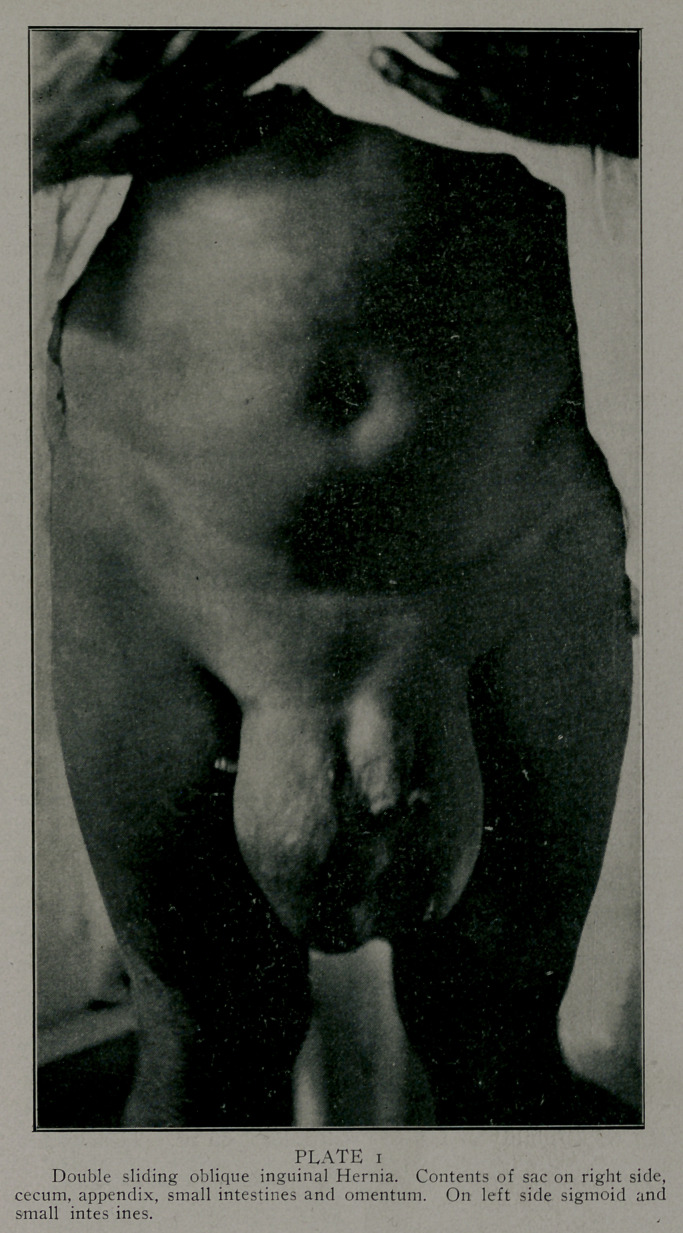


**PLATE 2 f2:**
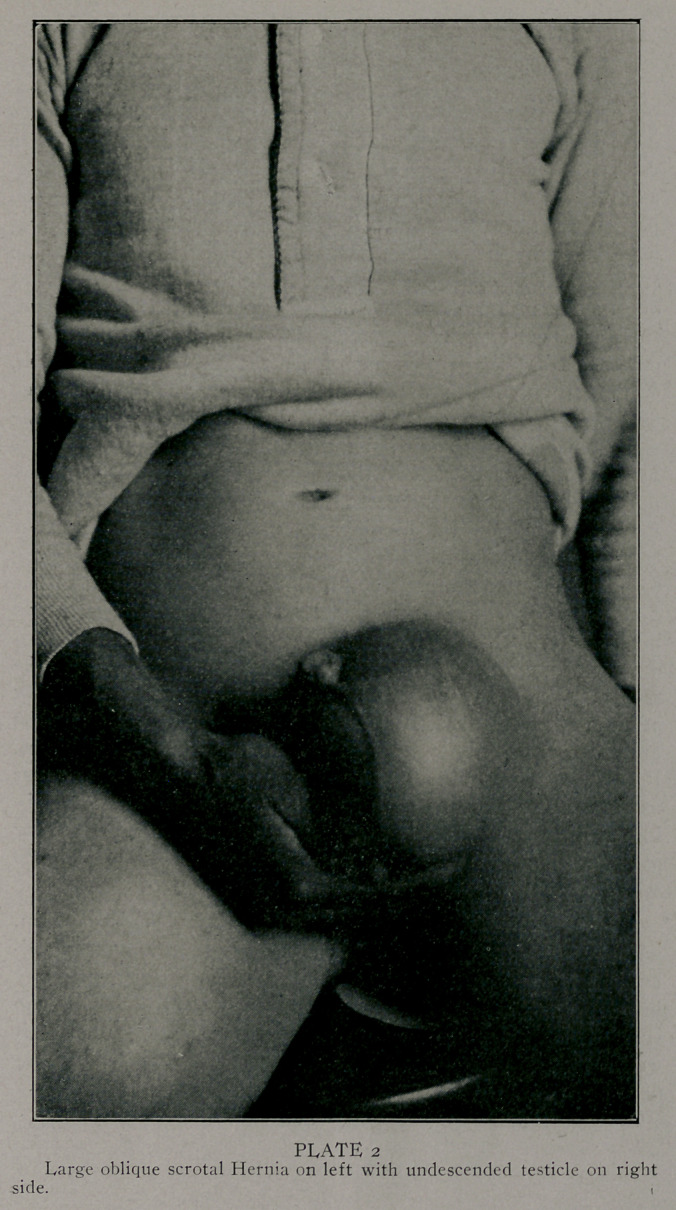


**PLATE 3 f3:**
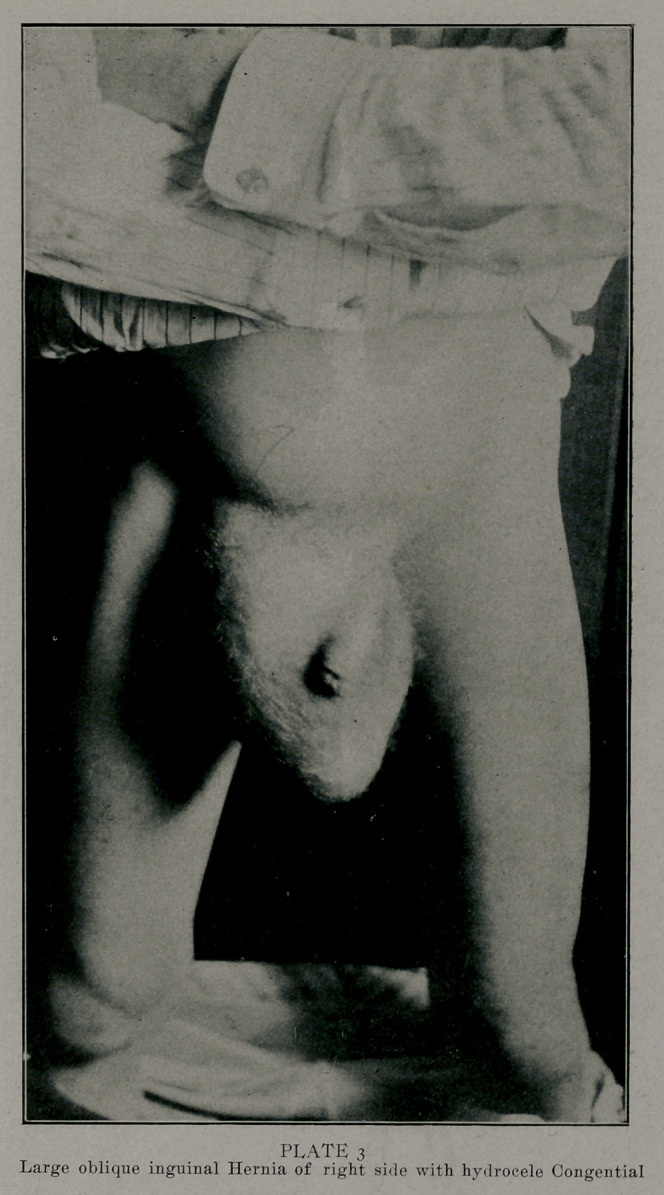


**PLATE 4 f4:**
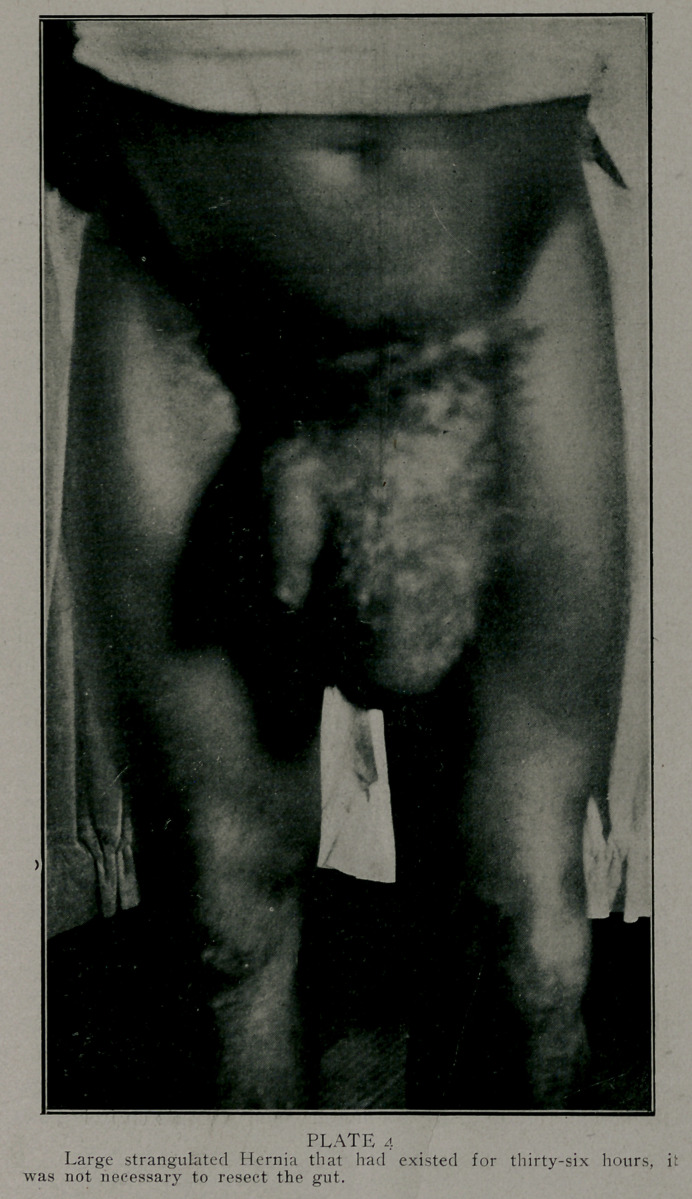


**PLATE 5 f5:**
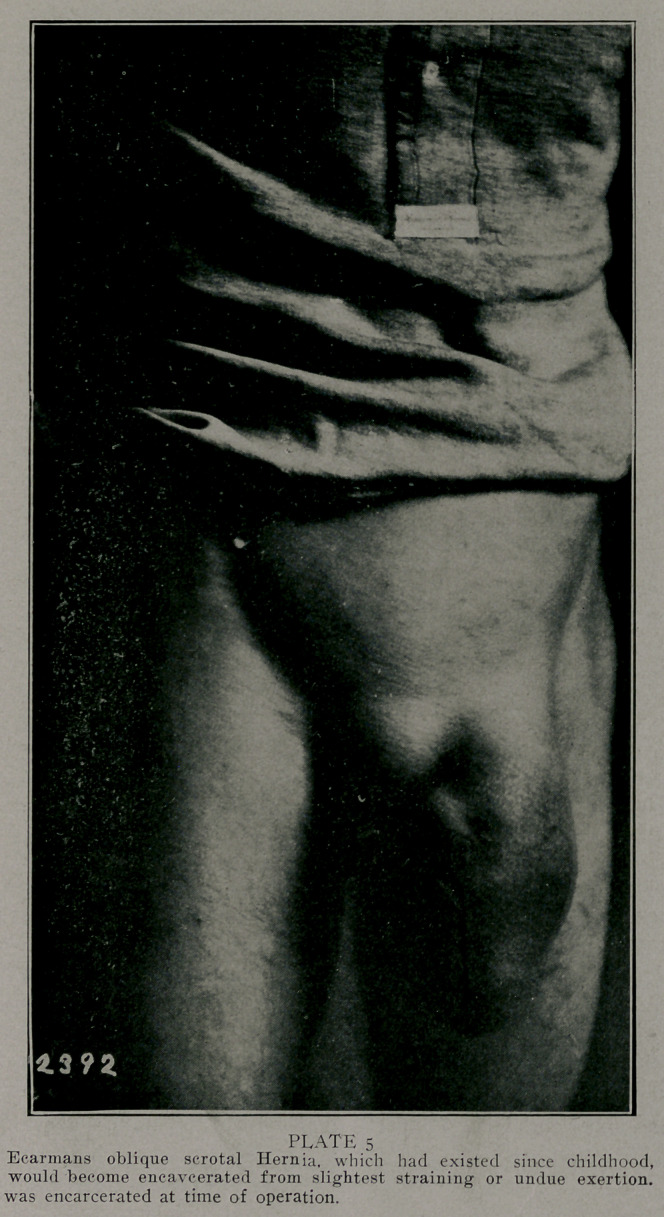


**PLATE 6 f6:**